# SY-707, an ALK/FAK/IGF1R inhibitor, suppresses growth and metastasis of breast cancer cells: SY-707 is an ALK/FAK/IGF1R inhibitor

**DOI:** 10.3724/abbs.2022008

**Published:** 2022-02-10

**Authors:** Ping Liu, Yinghui Sun, Shuang Liu, Jing Niu, Xijie Liu, Qiaoyun Chu

**Affiliations:** 1 Department of Geriatrics Xinhua Hospital Affiliated to Shanghai Jiaotong University School of Medicine Shanghai 200092 China; 2 Department of Biochemistry and Molecular Biology School of Basic Medical Sciences Capital Medical University Beijing 100069 China; 3 Shouyao Holdings Co. Ltd Beijing 100195 China

**Keywords:** SY-707, anti-tumor, anti-metastasis, breast cancer

## Abstract

Focal adhesion kinase (FAK), a multi-functional cytoplasmic tyrosine kinase, plays a critical role in cancer migration, proliferation and metastasis via regulating multiple signaling pathways. SY-707 is an anaplastic lymphoma kinase (ALK)/FAK/type 1 insulin-like growth factor receptor (IGF1R) multi-kinase inhibitor which is now being evaluated in phase II clinical trials for ALK positive non-small cell lung cancer (NSCLC). However, the effect of SY-707 on breast cancer is unknown. In this study, we assessed preclinical the anti-growth and anti-metastasis potency of SY-707 in breast cancer cells. ATP content, PE-Annexin V, and would healing assays were used to examine cell proliferation, cell cycle and migration. Then, SD rat and beagle dog models were used to evaluate the pharmacokinetics profile of SY-707, and mouse xenograft model was used to evaluate the anti-cancer activities of SY-707 *in vivo*. We found that breast cancer cells apoptosis were induced by SY-707. Moreover, SY-707 exerted inhibition on cell migration and adhesion in a dose-dependent manner. In T47D xenograft mice, SY-707 had significant anti-tumor activities alone or synergistically with Paclitaxel. Meanwhile, SY-707 also displayed significant suppression on spontaneous metastasis of tumor to the lung in 4T1 murine breast cancer xenograft model. In conclusion, SY-707 has potent anti-proliferation and anti-migration potential in breast cancer *in vitro* and *in vivo*, implying its therapeutic application for the treatment of breast cancer in future clinical trials.

## Introduction

Breast cancer is the most common diagnosed cancer for women worldwide. It is estimated that more than 1.4 million women worldwide are diagnosed with breast cancer, and over 450,000 women will die from this disease [1]. Treatment of breast cancer is particularly difficult when cancer metastasis occurs in other organs spreading from primary lesions [2]. In the past several decades, although new diagnostic, prognostic and therapeutic strategies of breast cancer were developed, the survival rate for breast cancer patients with metastatic disease has not changed significantly [3].

FAK, an intracellular non-receptor protein tyrosine kinase with a molecular weight of 125 kDa, performs its biological functions by interacting with multiple cytokine factors such as integrins, IL-4, VCAM-1, as well as growth factor receptors like epithelial growth factor receptor (EGFR), vascular endothelial growth factor receptor (VEGFR), and platelet-derived growth factor receptor (PDGFR) [4]. FAK also works as a nuclear protein to promote degradation of oncogene proteins p53 via ubiquitination and enhanced cell proliferation and reduced inflammatory responses [5]. Moreover, FAK has been implicated in the development of breast cancer and other malignancies. High level of FAK expression was observed in aggressive breast cancer [ 6, 7], and only low expression of FAK protein was detected in normal human breast tissue and para-carcinoma tissue [8]. Thus, FAK can be a marker of malignant transformation and a prognostic indicator in breast cancer. Recently, accumulating evidence supported that FAK is a therapeutic target for cancer treatment [ 9– 12], and several FAK inhibitors including PF-562271 [ 13, 14] and TAE226 [15] were approved for clinical studies. However, no positive clinical results have been released and efficacious FAK inhibitors are expected for further development [16].

SY-707, previously known as CT-707, is a multiple kinase inhibitor against ALK, FAK and IFG1R, which was approved for the treatment of ALK-positive patients with NSCLC in 2016 (NCT02695550). As a representative of independently developed Chinese medicine, SY-707 is expected to reduce the medical burden of patients and become a second-generation ALK-targeting drug with great market potential. However, there are no studies on the effect of SY-707 on breast cancer.

In this study, we analyzed the anti-tumor and anti-metastasis effects of SY-707 on breast cancer *in vitro* and *in vivo*, and the results will expand the clinical indications of SY-707 in the future.

## Materials and Methods

### Compound synthesis

N-isopropyl-2-((2-((2-methoxy-4-(4-(4-methylpiperazin-1-yl)piperidin-1-yl)phenyl)amino)-6,7-dihydro-5H-pyrrolo[2,3-d]pyrimidin-4-yl)amino)benzenesulfonamide (SY-707) was provided by Centaurus BioPharma (Beijing, China). PF-562271 was purchased from Selleckchem (Houston, USA). Compounds were stocked at 10 mM in 100% DMSO and diluted for biological test prior to use in each experiment.

### Kinase enzyme assay

Homogeneous time-resolved fluorescence (HTRF) assay was performed to analyze kinase enzyme activity and compound potency using HTRF ^®^ KinEASE ^TM^-TK kit (Cisbio Bioassays, Shanghai, China). Compound SY-707 was serially diluted by 3 folds from 1 mM stock solution in DMSO, and 4 μL of the diluted solution of the compounds was added to 96 μL of reaction buffer (50 mM HEPES, pH 7.0, 0.1 mM Na _3_VO _4_, 0.01% BAS, 5 mM MgCl _2_, 1 mM DTT), then 2.5 μL of 4× compound solution and 5 μL of 2× kinases solution were added to a 384-well plate (OptiPlate-384; PerkinElmer, Wellesley, USA ). The resulted solutions were briefly mixed, centrifuged and incubated for 5 min. Finally, 2.5 μL of 4× ATP/TK peptide (ATP at *K*_m_ for each kinase) solution was added to the reaction system to initiate the reaction. The enzymatic reaction was carried out for 60-120 min at 23°C, then terminated by addition of 5 μL of detection solution containing TK antibody-cryptate and 5 μL of Streptravidin-XL-665. The mixture was incubated for additional 1 h at 23°C. The fluorescent signals were measured with an EnVision multilabel plate reader (PerkinElmer). IC _50_ values of the compounds were calculated using the GraFit software (Version 6.0) and presented as the mean of three independent experiments performed in duplicate.

### Cell proliferation assay

MCF-7, T47D, MDA-MB-231, MIA Paca-2, PANC-1, SW620, HT-29, U87MG, and A549 cell lines were purchased from the Cell Bank of Chinese Academy of Science (Shanghai, China). Cells were cultured in DMEM or RPMI-1640 (Gibco, BRL, USA) supplemented with 10% FBS (HyClone, Logan, USA) and 50 IU penicillin/streptomycin in a humidified atmosphere with 5% CO _2_ at 37°C. The effects of SY-707 on cell proliferation were measured using CellTiter-Glo ^®^ or CellTiter-Blue ^®^ Cell Viability Assay kits (Promega, Beijing, China). In brief, cells were seeded in 96-well plates at low destiny with 195 μL of medium per well. The stock solutions of compounds were 3-fold serially diluted with DMSO from 10 mM to 0.3 nM, and 4 μL of solution at each concentration was mixed with 96 μL of serum free medium, then 5 μL of the resulted solution was added to each well. After treatment for 72 h, 25 μL of CellTiter-Glo ^®^ reagent (for adherent cells) or 35 μL CellTiter-Blue ^®^ reagent (for suspension cells) was added to each well, and the resulted mixture was further incubated at room temperature for 10 min or 4 h. The luminescence signals were measured using EnVision ^®^ multilabel plate reader (PerkinElmer), and the fluorescent signals were examined using FlexStation 3 (Molecular Devices, Sunnyvale, USA), and the IC _50_ values were calculated using Prism ^®^ software (Version 5.0) and presented as the average of three independent experiments performed in duplicate.

### Western blot analysis

MCF-7 and T47D cells were collected and lysed in lysis buffer (1% NP-40, 20 mM Tris-HCl, pH 8.0, 137 mM NaCl, 10% glycerol, 1 mM sodium orthovanadate, 1 mM PMSF, phosphatase inhibitors, and protease inhibitors), and total protein concentrations were determined using a BCA ^®^ kit (232225; Thermo Fisher Scientific, Waltham, USA). Equal amounts of cell lysates were loaded onto 10% SDS-PAGE gel and separated by electrophoresis. Separated proteins were then electro-transferred onto polyvinylidene fluoride (PVDF) membranes (Millipore, Bedford, USA). After being blocked with Tris-buffered saline containing 0.1% Tween-20 (TBST) and 5% bovine serum albumin (BSA), the membranes were incubated with primary antibodies at room temperature for 2 hours or at 4°C, then washed with TBST, followed by treatment with horseradish peroxidase (HRP)-conjugated goat anti-rabbit IgG secondary antibody at room temperature for another 1 h. The targeted protein bands were visualized using an enhanced chemiluminescence (ECL) plus system (Thermo Fisher Scientific). Primary antibodies against phospho-FAK (3283, 1:1000 dilution), phospho-ERK (4377, 1:2000 dilution), phospho-AKT (4060, 1:2000 dilution), FAK (3285, 1:1000 dilution), AKT (6703, 1:1000 dilution) and β-Actin (5125, 1:10,0000 dilution) were purchased from Cell Signaling Technology (Beverly, USA) and the HRP-conjugated goat anti-rabbit IgG (ZDR-5306, 1:10,000 dilution) was purchased from Jackson (West Grove, USA ).

### Flow cytometric analysis

MCF-7 cells were treated with 0.1% DMSO (control) or with SY-707 solution at indicated concentrations for 24 h at 37°C, then the cells were harvested by centrifugation at 350 *g* and washed twice with PBS. Cell apoptosis was determined using the PE Annexin V Apoptosis Detection kit (BD Biosciences, San Jose, USA) following the manufacture’s guides. Breifly, cells were resuspended with 100 μL of binding buffer containing Annexin V and 7-AAD. After 15 min of incubation at room temperature in the dark, 400 μL of binding buffer was added to the mixture and the cells were analyzed with a Guava flow cytometer (Millipore). Cell cycle arrest was evaluated by incorporation of propidium (PI; Sigma-Aldrich, St Louis, USA) into the permeablized cells. After being treated with DMSO or compounds for 24 h, cells were harvested, washed twice with cold PBS, fixed with ice-cold 70% ethanol, followed by a secondary staining step with staining buffer (0.25 mg/mL RNase, 0.025 mg/mL PI, in PBS) for 1 h at 37°C. Compound regulation of cell-cycle was analyzed using the Guava flow cytometer. Data were analyzed with FlowJo software.

### Would healing assay

Totally, 5×10 ^5^ cells were plated into each well of 6-well plates, and cultured to 70%–80% confluence, then the cell monolayer was gently and slowly scratched with a new 1-mL pipette tip across the center of the well. After scratching, the wells were gently washed with PBS twice to remove the detached cells, and replenished with fresh medium containing indicated concentration of compounds. The treated cells were allowed to grow for additional 24 h, and images were capturedunder a microscope. The migration distance were calculated and analyzed to evaluate the effects of compounds.

### Cell adhesion assay

MCF-7 and T47D cells were plated in 6-well plates and pretreated with different concentrations of PF-562271 or SY-707 for 24 h, and then trypsinized, washed and resuspended in culture medium. Cells were added into each well and incubated for 6 h. Non-adherent cells were collected by washing with PBS and counted.

### Tumor xenograft studies in nude mice

Female athymic nude mice (5–6 weeks old) purchased from the China Agricultural University Veterinary Teaching Hospital (Beijing, China) were used for all *in vivo* studies. A total of 5×10 ^6^ or 5×10 ^5^ of T47D or 4T1 cells in 100 μL of serum-free medium were injected subcutaneously into the right and left flanks of mice. Tumor volumes were monitored by caliper measurement using the formula: *v* (mm ^3^) = ( *w* ^2^ × *l*)/2, where *v* is the tumor volume, *w* is width and *l* is length. When the tumor volumes reached 150–200 mm ^3^, mice were randomized into treatment and control groups (10 mice per group) and treated with compounds or vehicle by oral gavage every day for 2–4 weeks. The tumor volumes and body weight were measured every 2–3 days, and tumor growth inhibition (TGI) and body weight changes were calculated. All the experiments were carried out in accordance with the Guidelines of Institutional Animal Care and Use Committee (IACUC) of Shouyao Holdings and approved by IACUC of Shouyao Holdings.

### Metastasis analysis *in vivo*

Metastasis was detected in mouse lungs after the mice were sacrificed at the last dosing day. The lungs of each mouse were excised immediately and fixed with 4% paraformaldehyde for 24 h, then the lungs were photographed (three photographs per section per mouse) and the metastatic nodules in the lungs were manually counted and the data was analyzed for compound evaluation.

### Pharmacokinetic studies

Female SD rats (5-6 weeks old) or beagle dogs were administrated with SY-707 at a single dose of 5 mg/kg, and blood samples were taken sequentially from 2 min to 24 h after compound administration. Animals were purchased from the China Agricultural University Veterinary Teaching Hospital (Beijing, China), and the study was approved by the institutional animal care and use committee of Shouyao Holdings. The serum samples were collected by centrifugation and stored at –80 ^o^C. Compound concentrations in the sample serums were further analyzed with an API-4000 LC-MS/MS system (Applied Biosystem, Foster City, USA), and pharmacokinetic parameters ( *T*_1/2_, *T*_max_, *C*_max_, *AUC*_0-INF_, Cl_F_obs, MRT _INF_obs_, and F(%)) were calcuated using the WinNonlin software (version 6.2; WinNonlin Professional, Pharsight, USA).

### Statistical analysis

Data were presented as the mean±standard deviation (SD). Two-tailed *t* test was performed to compare the difference between two groups. *P*<0.05 was regarded to be statistically significant.

## Results

### SY-707 is a multi-kinase inhibitor against ALK, FAK and IFG1R

**Figure 1:**
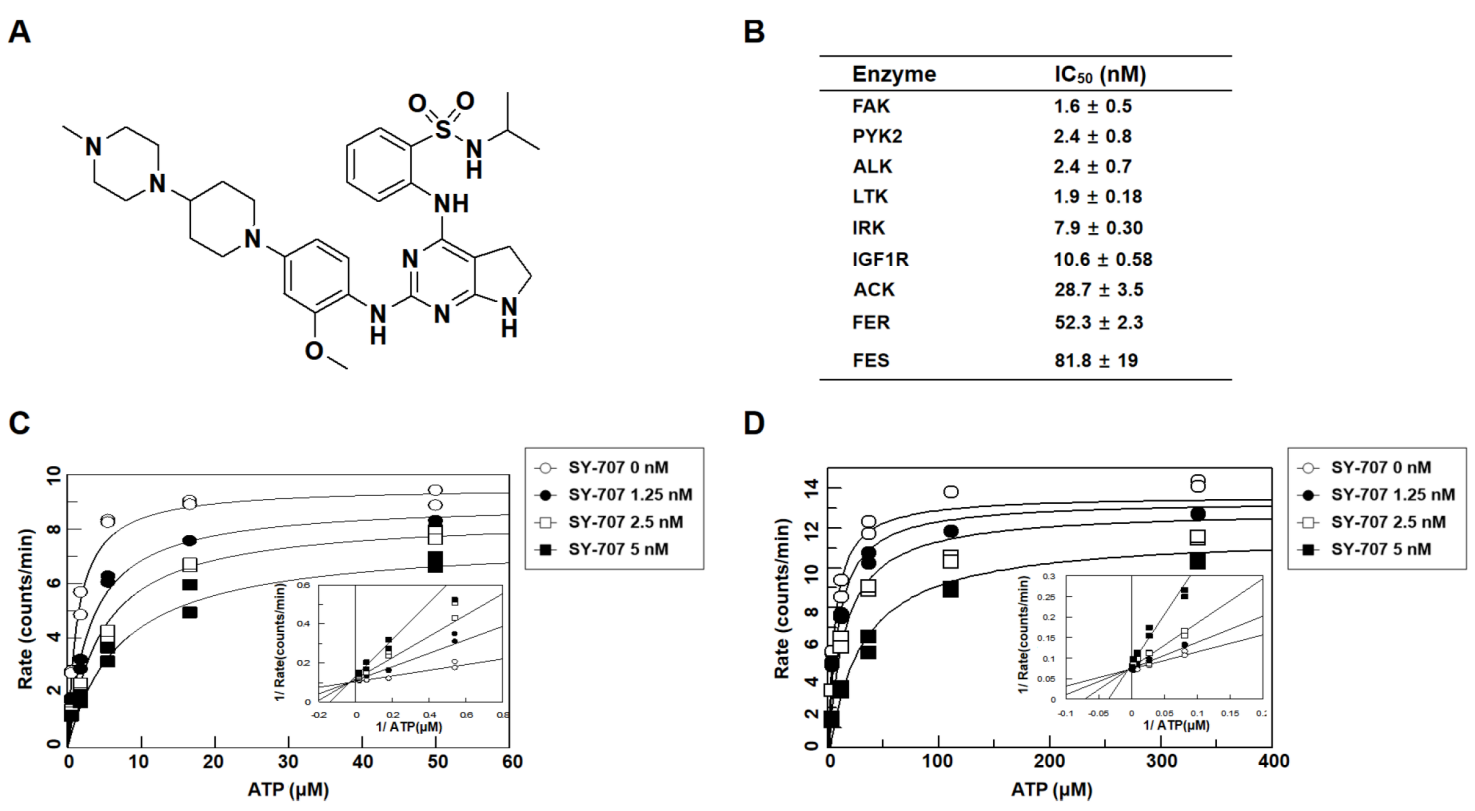
Figure1 ***In vitro* enzymatic inhibition activity of SY-707 against multiple kinases **(A) Chemical structure of SY-707. (B) Kinase inhibition profile of SY-707. (C) ALK enzyme activities were evaluated at various concentrations of ATP (0–50 μM) in the presence of different concentrations of SY-707. (D) FAK enzyme activities were evaluated at various concentrations of ATP (0–333 μM) in the presence of different concentrations of SY-707.

SY-707 ( Figure 1A) was originally developed as an ALK inhibitor. It showed very potent inhibition activity on ALK kinase with an IC _50_ value of 2.4 nM ( Figure 1B). To evaluate the *in vitro* enzymatic potency and selectivity of SY-707, a kinase panel screening on 96 kinases was performed to determine the inhibitory activities of SY-707 at a concentration of 1 mM. The results showed that SY-707 was able to inhibit ALK as well as several other kinases, such as FAK, Pyk2, LTK, IRK and IGF1R, markedly with IC _50_ values around 1–10 nM ( Figure 1B). Then, enzymatic kinetic analysis of ALK and FAK was performed to explore the mechanisms of inhibition effect of SY-707. Activities of ALK or FAK were estimated at various concentrations of ATP in the presence or absence of SY-707. It was found that SY-707-mediated inhibition of ALK kinase activity can be significantly attenuated with increasing concentration of ATP ( Figure 1C). SY-707 had higher affinity to free ALK enzyme, with a dissociation constant ( *K*_i_) value of 0.90 nM, than the ALK enzyme-ATP complex, with a *K*_i_′ value of 19.2 nM. Lineweaver-Burk plots (double-reciprocal plots of 1/ *v* versus 1/[ *s*]) (inset of Figure 1C) illustrated that the four plots were intersected on *Y* axis, indicating that SY-707 inhibited ALK kinase as an ATP-competitive inhibitor. Similarly, SY-707 also preferred to bind with free FAK enzyme, with a dissociation constant ( *K*_i_) value of 0.56 nM, rather than to bind with the FAK enzyme-ATP complex, with a *K*_i_′ value of 16.1 nM. And the four double-reciprocal plots were also intersected on *Y* axis, suggesting an ATP-competitive inhibitory behavior of SY-707 for FAK kinase (inset of Figure 1D). Since there is sequence similarity between catalytic domains of multiple kinases, it is reasonable that SY-707 inhibits multiple kinases in an ATP-dependent manner.

### SY-707 inhibits the proliferation of breast cancer cells

**Figure 2:**
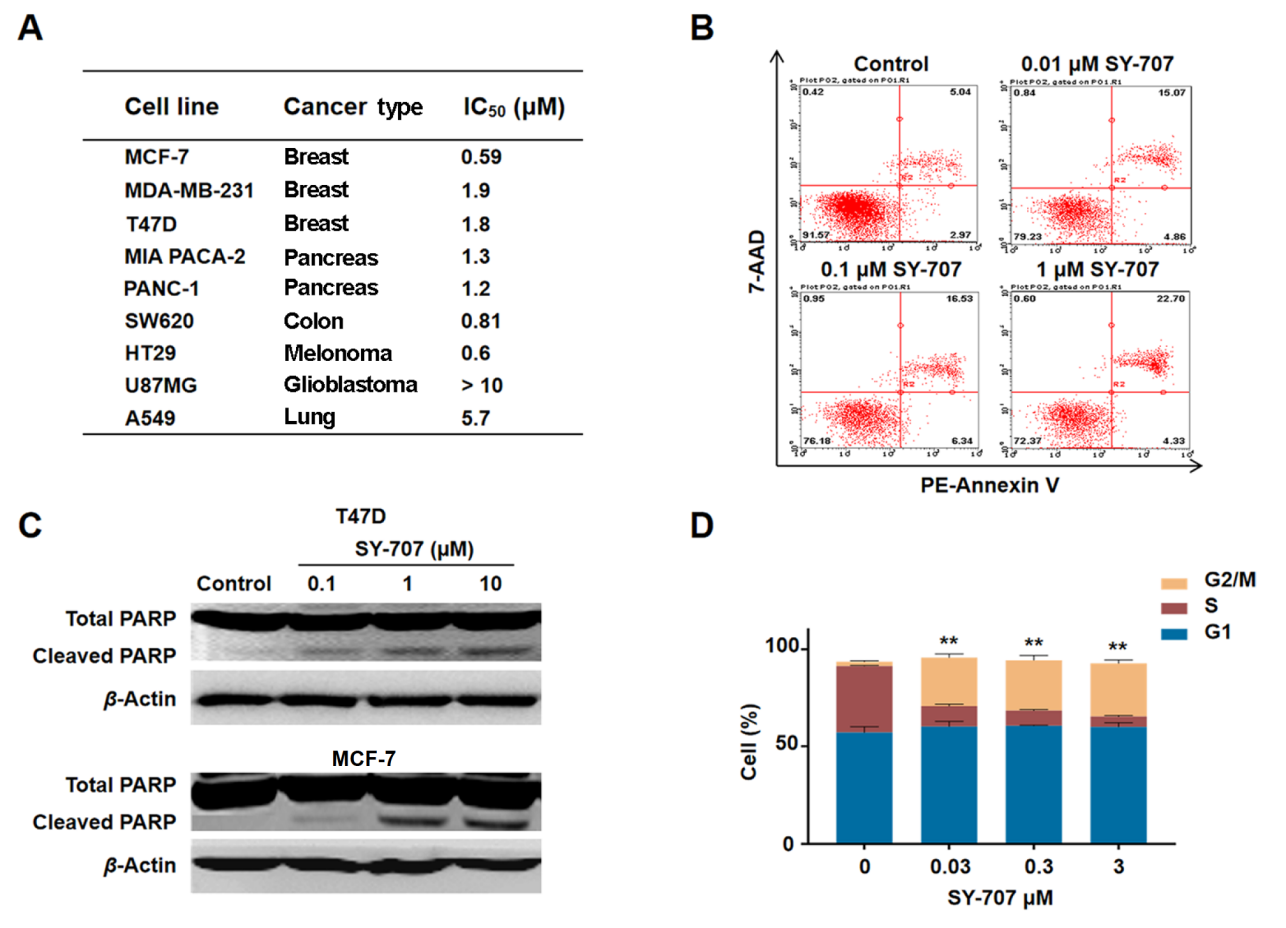
Figure2 **Effects of SY-707 on cell growth, cell apoptosis and cell cycle**(A) Anti-proliferation activities of SY-707 in a series of tumor cells. (B) Apoptosis of MCF-7 cells treated with SY-707. Cells were cultured and treated with SY-707 under the indicated concentrations (0.01, 0.1 and 1 μM respectively) for 24 h, then stained with Annexin-V and 7-AAD for cell apoptosis analysis by Guava flow cytometry. (C) T47D (upper) or MCF-7 (down) cells were incubated with various concentration of SY-707 for 24 h, then the cells were collected for western blot analysis using anti-PARP antibody. (D) Cell cycle of MCF-7 cells treated with SY-707. Cells were harvested and washed with PBS for three times after incubation with the specified concentration of SY-707 for 24 h, and cells were then labeled with propidium (PI) after fixing with ice ethanol. Cell cycle arrest was determined by Guava flow cytometry. **P<0.01.

To evaluate the cellular anti-tumor activities of SY-707, cell proliferation studies were carried out to evaluate the inhibitory effects of SY-707 on a series of cell lines including breast cancer (MCF-7, MDA-MB-231, T47D), pancreas cancer (MIA Paca-2, PANC-1), glioblastoma (U87MG), colon cancer (SW620), melanoma carcinoma (HT29), and lung cancer (A549). SY-707 significantly suppressed the growth of these solid cancer cells with IC _50_ values of 0.5–10 μM ( Figure 2A). Late apoptosis was dose-dependently induced by SY-707 in MCF-7 cells ( Figure 2B). The cleaved poly-ADP ribose polymerase (PARP) was markedly detected in these SY-707-treated cells and the amount of cleaved PARP was correlated with the concentration of SY-707 ( Figure 2C), meaning that SY-707 induces cell apoptosis by the formation of active apoptotic executor PARP. Meanwhile, SY-707 was able to induce pronounced G2/M phase arrest and a decrease in the percentage of cells in S phase in a dose-dependent manner in MCF-7 cells ( Figure 2D). These data indicated that SY-707 potently attenuated cell growth via blocking cell cycle progression and inducing active PARP to execute apoptosis in breast cancer cells.

### SY-707 blocks the migration and adhesion of breast cancer cells

**Figure 3:**
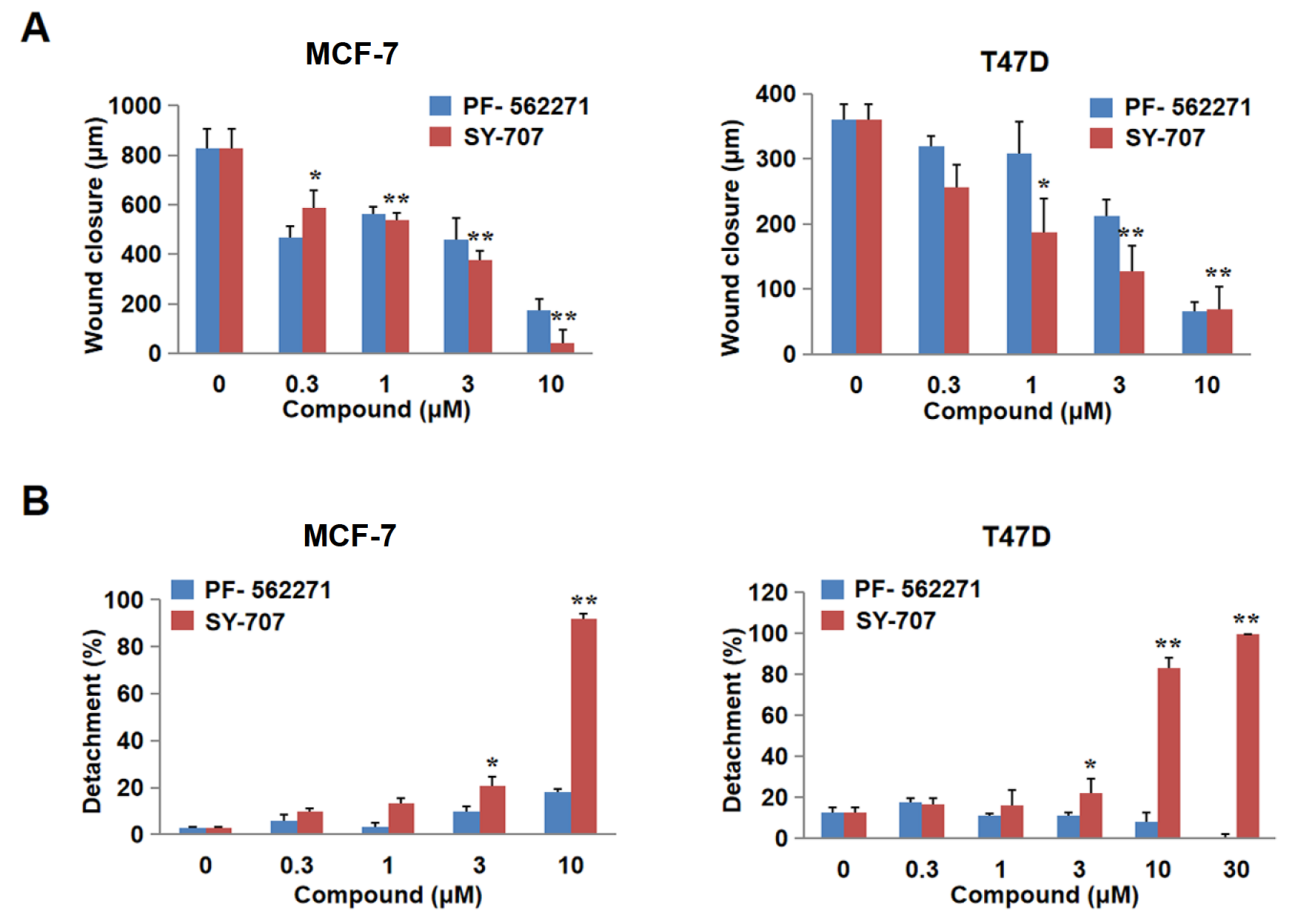
Figure3 **SY-707 blocks cell migration and adhesion**(A) The migration distances of MCF-7 and T47D cells were calculated after the cells were incubated with SY-707 for 24 hours. (B) Effects of SY-707 on cell adhesion in MCF-7 and T47D cells. The cells were plated into 6-well plates, mixed with SY-707, and then the number of unattached cells was counted after washing. *P<0.05, **P<0.01.

As FAK plays key roles in cancer migration and metastasis, the effects of SY-707 on cell invasion and cell adhesion were evaluated. It was found that migration distance was significantly decreased in SY-707 treatment group compared to that in the control group in a dose-dependent manner. Meanwhile, significant reduction of migration in MCF-7 and T47D cells was also observed in PF-562271-treated group ( Figure 3A). At the same time, SY-707 characteristically repressed the adhesion of MCF-7 and T47D cells in a dose-dependent manner ( Figure 3B). These data indicated that SY-707 suppresses cell invasion and cell adhesion of breast cancer cells.

### SY-707 suppresses FAK and IGF1R signaling pathway in breast cancer

**Figure 4:**
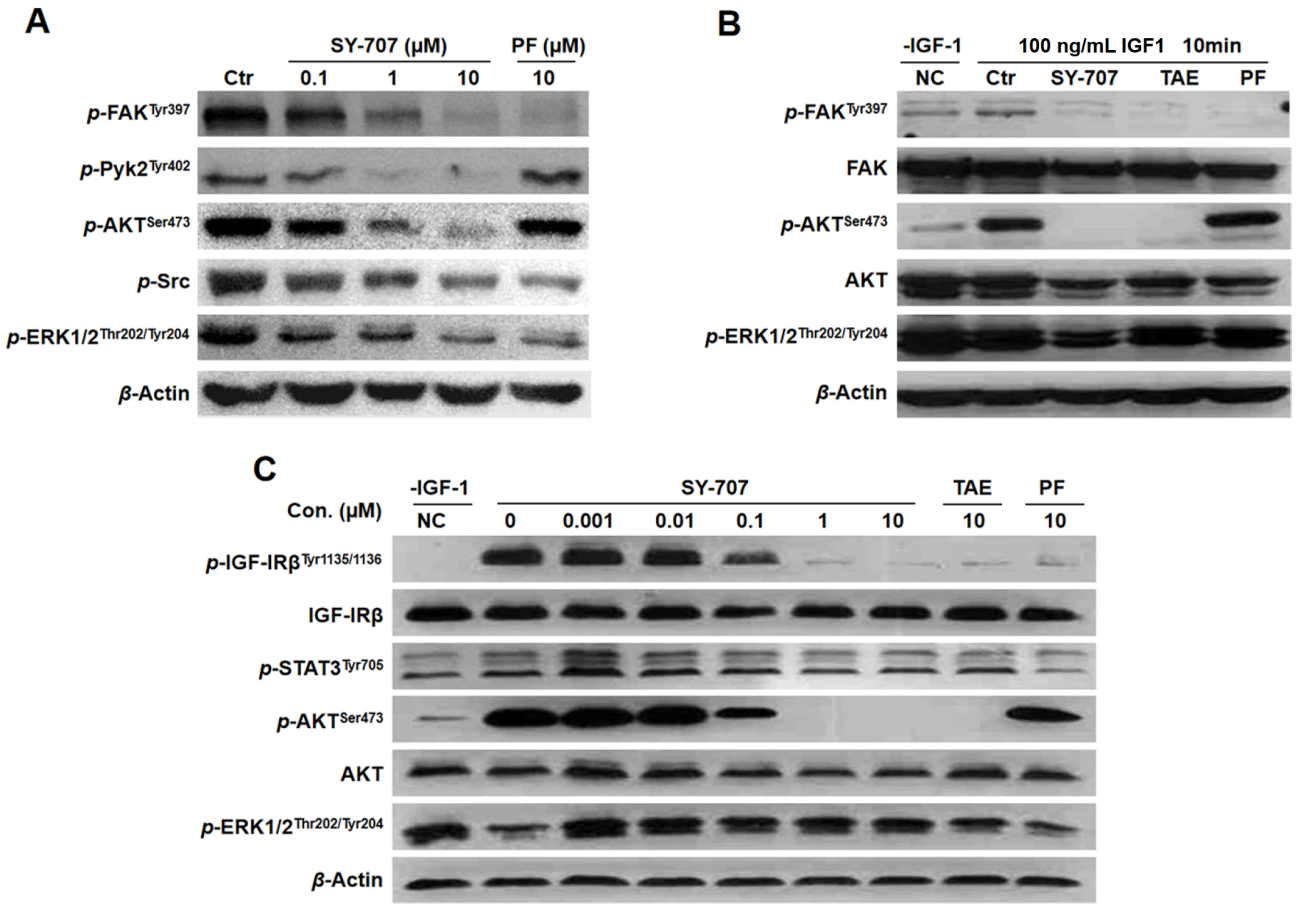
Figure4 **Inhibitory effects of SY-707 on the phosphorylation of FAK**(A,B) Concentration-dependent inhibition of SY-707 on phosphorylation of FAK in T47D (A) and MCF-7 (B) cells. Cells were cultured in 6-well plates and treated with the indicated concentrations of SY-707 for 1 h before stimulation with or without 100 ng/mL IGF1 for 10 min. Control cells received the drug vehicle with 0.1% DMSO. Cell lysates were analyzed by western blot analysis using specific antibodies against p-FAK, p-Pyk2, p-AKT and p-ERK respectively. (C) Concentration-dependent inhibition of SY-707 on phosphorylation of IGF1R in T47D cells. The cells were incubated with SY-707 for 1 h before stimulation with 100 ng/mL IGF1 for 10 min. Control cells received the drug vehicle with 0.1% DMSO. Cell lysates were analyzed by western blot analysis using specific antibodies against p-IGF1R, p-STAT3, p-AKT and p-ERK respectively.

To investigate the underlying mechanisms of SY-707 inhibition on cell proliferation, we evaluated the effects of SY-707 on FAK and IGF1R signaling pathways that play important roles in the proliferation of several types of cells based on its kinase targets. Since autophosphorylation at Tyr397 is an important event for FAK to maintain the biological function, western blot analysis was performed to determine the phosphorylation levels of FAK and downstream proteins. SY-707 dose-dependently suppressed the phosphorylation of FAK at Tyr397 and Pyk2 at Tyr402 in T47D and MCF-7 cell lines after these cells were treated with SY-707 for 1 h ( Figure 4A,B), and the phosphorylation of downstream proteins, including AKT and ERK, was also decreased following the blockade of FAK and Pyk2 phosphrylation. More importantly, SY-707 exhibited higher potency than reference compounds PF-562271 and TAE226 in parallel. Meanwhile, SY-707 was able to block the phosphorylation of IGF1R in T47D cells in a dose-dependent manner ( Figure 4C) after stimulation with the ligand IGF1 for 10 min. The phosphorylation of IFG1R was significantly suppressed at lower concentration of SY-707, competitively to PF-562271 and TAE226. Similarly, the phosphorylation of downstream kinases AKT and ERK was decreased subsequently. These results implied that SY-707 regulates FAK and IGF1R signal transduction pathways to inhibit tumor cell growth and tumorgenesis via inhibiting the activities of these two kinases, meaning that proliferation-associated MAPK and AKT signaling pathways are keys of SY-707’s inhibition of the growth of FAK- or IGF1R-expressing cells.

### SY-707 has anti-tumor activity *in vivo* via FAK inhibition

Since SY-707 showed excellent *in vitro* potency in enzymatic and cellular assays, we further evaluated its pharmacokinetics profile in SD rats and beagle dogs by oral (peros, PO) before *in vivo* efficacy studies. SY-707 was eliminated slowly in SD rats or beagle dogs, with a *t*_1/2_ value of 12.1 h and 9.72 h respectively, and showed high level in the plasma, with a mean AUC _0-INF_ of 10,964 h∙ng/mL for rats and 459 h∙ng/mL for beagle dogs correspondingly ( Table 1). In addition, SY-707 also had good clearance parameters and oral bioavailability in these two species. The distribution of SY-707 in rat tissues (AUC 0–24 h) was as follows: uterus, spleen, skeletal muscle, heart, lung, body fat, testis, liver, stomach and intestine, kidney, ovary and brain. Except the uterus and spleen, other organs had lower level of SY-707 than the plasma ( Supplementary Figure S1).

**Table:** **Table1** Pharmacokinetics parameters of SY-707 treatment (5 mg/kg, PO) in SD rats and beagle dogs

Species	Rat		Beagle dog
Male	Female	All		Male	Female	All
t _1/2_ (h)	13	11.2	12.1		9.33	10.1	9.72
*T*_max_ (h)	3.33	4.33	3.83		3.00	2.67	2.83
*C*_max_ (ng/mL)	909	639	774		37.8	34.2	36
*AUC*_0-INF_ (h∙ng/mL)	13,335	8593	10,964		479	438	459
Cl_F_obs (mL/h/kg)	378	586	482		11,257	12,131	11,694
MRT _INF_obs_ (h)	15.5	13.8	14.7		13.6	13.7	13.6
F (%)	38.3	33.2	36.2		23.8	18.8	21.1

**Figure 5:**
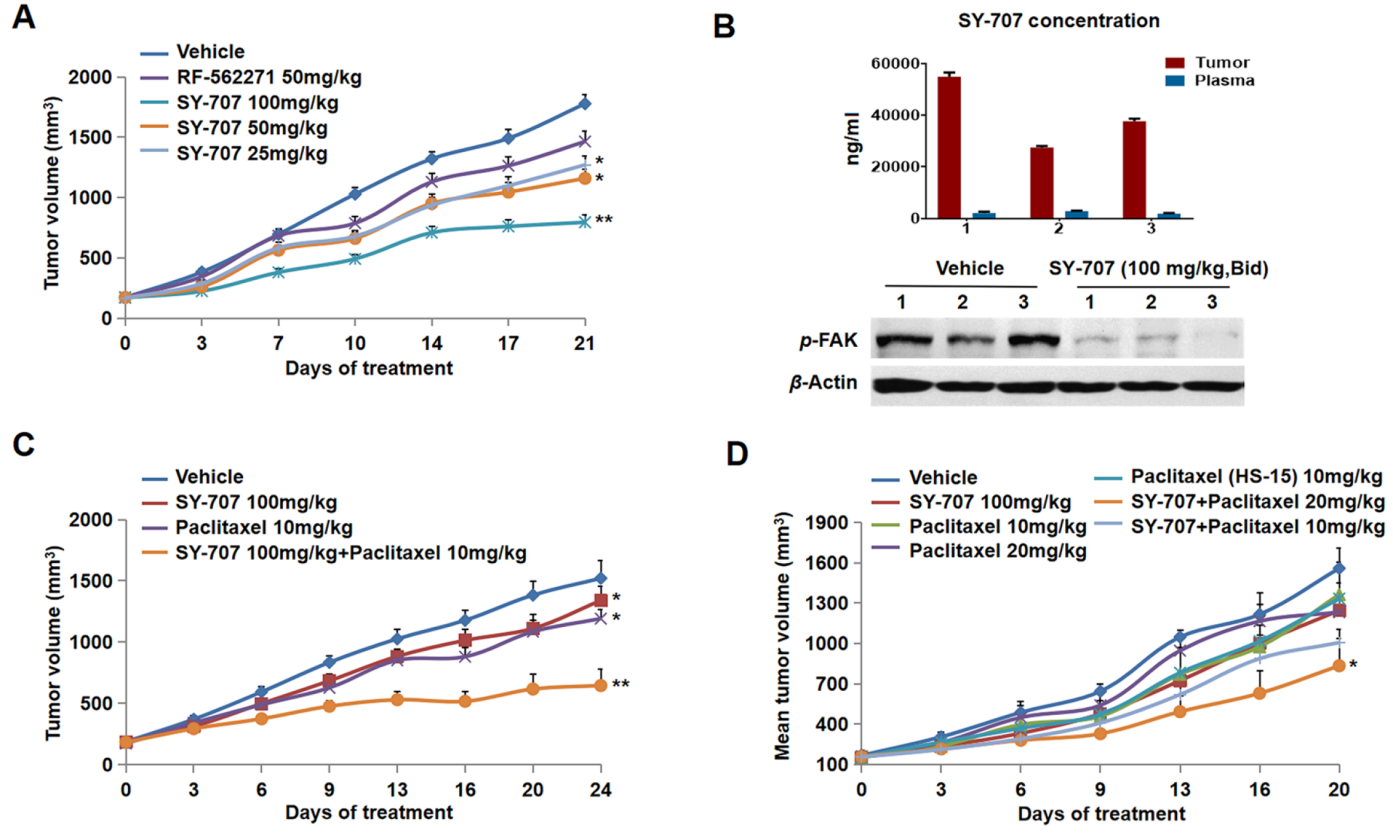
Figure5 **Anti-tumor activities of SY-707 in breast cancer xenografts**Xenograft tumors were established by inoculating T47D (A, B, C) cells or 4T1 (D) cells into the nude mice. Oral administration of SY-707 alone or combined with paclitaxel was initiated when tumors reached an average of approximately 150–300 mm3 in volume and continued through the experiment. Tumor volume was measured on the indicated days with the mean tumor volume±SD indicated for each group (10 mice/group). Each treatment group was compared to vehicle group with t-test. *P<0.05, **P<0.01. (B) The tumor tissues were harvested at 2 h after the last dosage of SY-707, compound levels in tumor tissues or plasma and the phosphorylation levels of FAK were analyzed.

Due to the strong cell growth inhibition potency of SY-707, we firstly evaluate its *in vivo* anti-cancer activity in T47D xenograft model mice. In this model, mice were orally administered twice a day with vehicle or SY-707 at dose of 100, 50 or 25 mg/kg after tumor size reached 150–300 mm ^3^. The results showed that SY-707 suppressed T47D tumor growth in a dose-dependent manner ( Figure 5A). After treatment with SY-707 for 21 days, tumor growth inhibition rate was estimated to be 61% ( *P*<0.01), 39% ( *P*<0.05), and 31% ( *P*<0.05) in the 100, 50, and 25 mg/kg group respectively, but only 20% in the reference compound PF-562217 group.

To explore the relationship between the *in vivo* anti-tumor growth activities and the inhibition on FAK signaling, pharmacokinetics/pharmacodynamics studies were performed in T47D xenograft model. The phosphorylation level of FAK at Tyr397 in SY-707 treatment groups were significantly decreased compared to that in the control group ( Figure 5B). Meanwhile, higher concentrations of SY-707 were also detected in tumor samples compared to that in the plasma, suggesting that SY-707 is able to reach the tumor tissues with high level.

Next, we evaluated the combination effects of SY-707 and paclitaxel (an approved drug for breast cancer therapy) in T47D xenograft model. Mice bearing T47D breast cancer were divided randomly into 4 groups: vehicle, SY-707 (100 mg/kg/day), paclitaxel (10 mg/kg/week), and SY-707 plus paclitaxel groups. After 24 days of treatment, tumor growth inhibition rate of xenograft mice in SY-707 (100 mg/kg/day) and paclitaxel (10 mg/kg/week) groups was 14% and 24% ( *P*<0.05), respectively, while tumor growth inhibition rate achieved 66% in the combination group ( *P*<0.01, Figure 5C).

In the 4T1 xenograft model, SY-707 was administered with or without paclitaxel at indicated concentration, and then anti-tumor activities were evaluated three times a week. Only slight suppression on 4T1 tumor growth was observed in all of monotherapy groups, while significant inhibition of tumor growth was detected in the combination group (100 mg/kg SY-707 plus 20 mg/kg paclitaxel) with a tumor growth inhibition rate of 51.5% ( *P*<0.05, Figure 5D). These data indicated that SY-707 had very potent anti-tumor activities in T47D xenograft models and sensitized *in vivo* efficacy of paclitaxel in T47D and 4T1 xenograft models, which revealed a direct relationship among SY-707 levels in tumor tissues, tumor growth inhibition rate, and the inhibition of phosphorylation of FAK.

### SY-707 represses spontaneous metastasis of breast cancer to the lung

**Figure 6:**
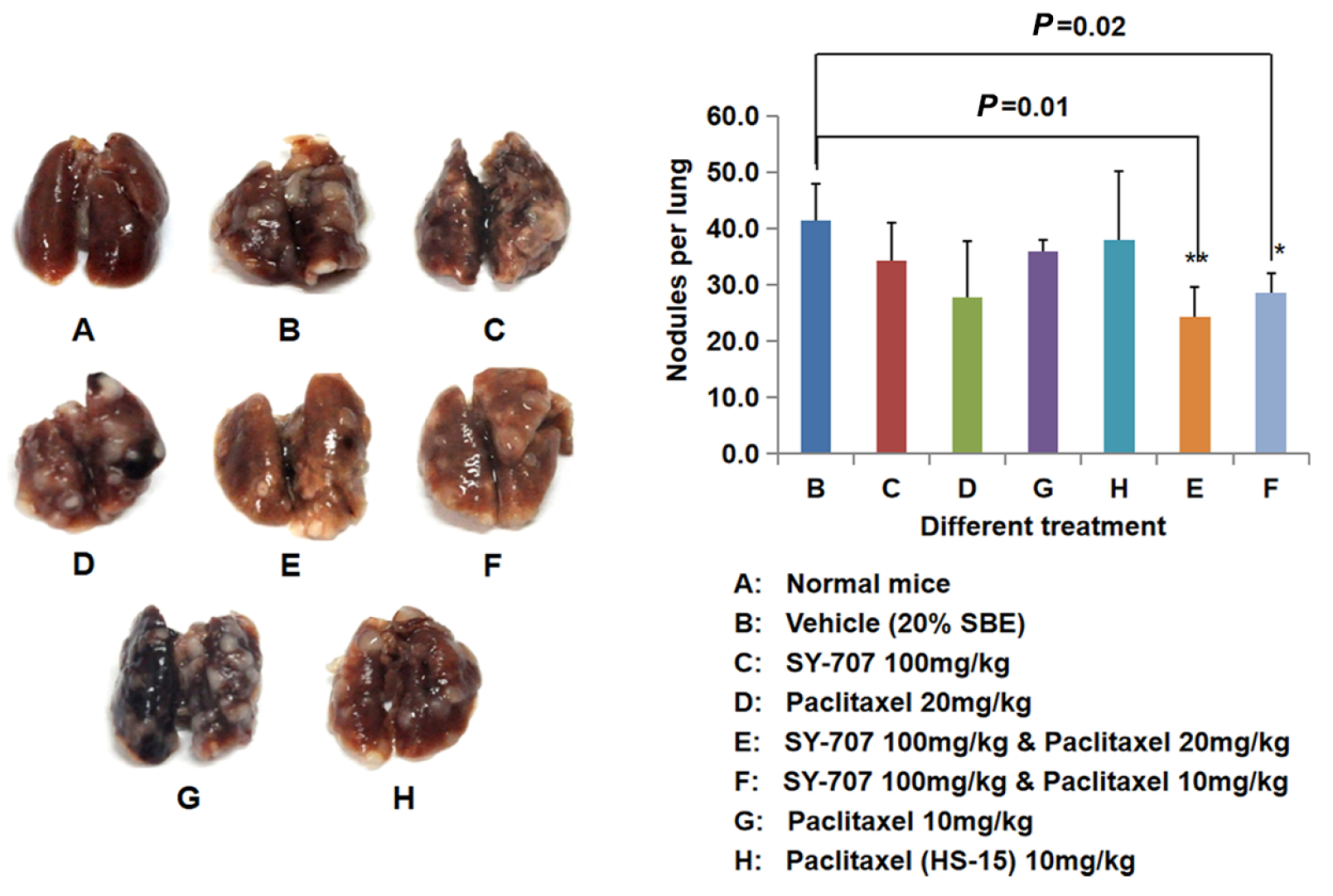
Figure6 **Anti-metastasis potency of SY-707 in mouse 4T1 model**Nude mice bearing 4T1 breast cancer was adminstered with SY-707 (100 mg/kg, PO, QD) / paclitaxel (20 or 10 mg/kg, IV, QW) alone (C, D, H and G) or combined (E and F) for 20 days (6 mice/group). The normal group (A) and the vehicle group which treatment with 20% SBE (B) were control. Mouse lungs were taken at the last day of treatment and fixed with 4% paraformaldehyde for 24 h, then the lungs were photographed and the metastatic nodules were counted manually for further analysis. *P<0.05, **P<0.01.

Cancer metastasis from primary area to other organs is a major cause of morbidity and mortality for breast cancer, since SY-707 displayed notable anti-migration activities in cellular assays, its *in vivo* anti-metastasis effects were evaluated in 4T1 xenograft model, in which the tumor metastasized to the lung spontaneously [17]. Mice were inoculated with 4T1 cells in PBS at right and left flanks expect the normal control group ( Figure 6A), SY-707 with or without paclitaxel was administered for 20 days when tumor volumes reached 150–300 mm ^3^. The mice were sacrificed on the last day of drug administration, then the lungs were collected and metastatic nodules were counted manually for the evaluation of metastasis of 4T1 breast cancer. Treatment alone with SY-707 at100 mg/kg/day (PO, peros), paclitaxel with 20% SBE or HS-15 at 10 mg/kg/week (IV, intravenous injection), or paclitaxel at 20 mg/kg/week (IV), reduced the number of metastatic nodules in the lungs compared to the vehicle group with 20% SBE ( Figure 6B), but not statistically different among the groups ( Figure 6C,D,G,H). In contrast, combination of SY-707 and paclitaxel was able to eliminate the number of metastatic nodules in nude mice bearing 4T1 breast cancer significantly. The number of metastatic nodules in the 100 mg/kg/day SY-707 plus 20 mg/kg/week paclitaxel group was decreased by more than 40% compared with that in the vehicle group ( *P*<0.01), and the number of metastatic nodules in the 100 mg/kg/day SY-707 plus 10 mg/kg/week paclitaxel group was also decreased by 31.1% compared with that in the vehicle group ( *P*<0.05) ( Figure 6E,F). These data suggested that SY-707 plus paclitaxel notably depressed cancer metastasis from the primary area to the lungs.

## Discussion

Development of kinase inhibitors is an efficacious approach in drug discovery for the treatment of multiple cancers, and has benefitted thousands of cancer patients [ 18– 20]. However, cancer metastasis makes most of the therapies fail because of the spread, invasion and relocation of metastatic cancer cells from primary areas to other organs in patients. Similarly, drug resistance and metastasis are the difficulties in the treatment of breast cancer. Several FAK inhibitors have entered the clinical stage (PF-573228, PF-562271, VS-6063, etc.), but no FAK inhibitors have been approved due to their insignificant efficacy or serious side effects. Therefore, discovery of anti-metastasis drugs is an attractive way to improve current therapy and to expand pipelines for many pharmaceutical companies.

SY-707 is a multi-kinase inhibitor targeting ALK, FAK and IFG1R for the treatment of ALK-positive NSCLC (including initial treatment and crizotinib resistance). Because of the excellent safety and efficacy shown in phase I clinical trials (Peking Union Medical College Hospital), SY-707 is currently undergoing phase II clinical trials in 39 hospitals in China. Thus, we systematically explored its inhibitory potency on cancer growth and cancer metastasis via suppressing FAK in breast cancer to explore additional indications for future clinical studies. SY-707 strongly suppresses tumor cell growth, which is well associated with the dose-dependent induction of cell apoptosis and cell cycle arrest at G2/M phase ( Figure 2D). PARP is a key DNA repair factor. Its inhibitors have been tested in BRAC1/2-mutant tumors and are in ongoing trials for the treatment of various malignancies including breast cancer. Cleaved PARP is released from apoptotic cells and used as a biomarker for the apoptotic response of tumor cells [21]. SY-707 promotes the formation of cleaved PARP ( Figure 2C). Overall, the results indicated that SY-707 potently attenuates cell growth via blocking cell cycle progression and inducing active PARP to execute apoptosis in breast cancer cells.

FAK is a key regulator of cell invasion, cell migration and cell proliferation of breast cancer [22]. SY-707 was originally developed as an ALK inhibitor with strong ALK kinase inhibition activity with an IC _50_ value of 2.4 nM. SY-707 also significantly inhibits other kinases, such as FAK, Pyk2, LTK, IRK and IGF1R, with IC _50_ values ranging from 1 to 10 nM. Among all the detected kinases, SY-707 markedly inhibits FAK in kinase assays ( Figure 1B). Meanwhile, we used western blot analysis to detect the effect of SY-707 on kinase protein levels, and subsequently explored the effects of SY-707 on FAK signaling cascades. We found that SY-707 depressed FAK signaling pathways via decreasing its phosphorylation and its downstream proteins ( Figure 4). More importantly, SY-707 is able to attenuate cell invasion and migration ( Figure 3), indicating that SY-707 may affect the biological functions via inhibiting FAK in cancer cells.

Further pharmacokinetic studies demonstrated that SY-707 had druggable pharmacokinetics parameters in SD rats and beagle dogs, such as slow metabolization (long *t*_1/2_ of 12.1 h in rat and 9.72 h in beagle dog) and high levels in the plasma (AUC of 10,964 h∙ng/mL in rats) ( Table 1 and Supplementary Figure S1). SY-707 displayed significant suppression activity on T47D or 4T1 breast cancer growth in animal models, and it had significant synergistic effects with paclitaxel. The inhibition of FAK phosphorylation in T47D breast cancer by SY-707 is well correlated with its concentrations in tumor tissues ( Figure 5B), indicating a possible mechanism-based blockage of *in vivo* tumor growth by SY-707. Indeed, the immunohistochemical results will definitely better prove the mechanism of SY-707 *in vivo*. In the future studies, the effects of SY-707 on more types of cancers will be explored.

Besides tumor growth inhibition, attenuation of cancer metastasis from primary region to the lungs was also observed in animal models in the SY-707 plus paclitaxel groups ( Figure 6), which is consistent with its cellular inhibitory potency on cancer cell invasion and attachment. Based on the current results, treatment with SY-707 possibly provides an approach to block cancer metastasis in future therapies for breast cancer. In particular, SY-707 has better pharmacokinetics parameters than known FAK and ALK inhibitor PF-562271, thus it should have better efficacy in clinical studies. Collectively, SY-707 could be developed as a multiple drug candidate for multiple indications including breast cancer besides ALK-positive NSCLC.

In conclusion, SY-707 is a multiple kinase inhibitor against several kinases including FAK, IGF1R, ALK, Pyk2 and others. It not only inhibits the growth of breast cancer, but also eliminates invasion of cancer *in vitro* and **in viv*o *, thus providing a therapeutic potential for the treatment of metastatic breast cancer and expanding additional indications in future clinical studies.

## Supporting information

446FigS1

## Supplementary Data

Supplementary data is available at *Acta Biochimica et Biophysica Sinica* online.
