# Supplementary material for: SY-707, an ALK/FAK/IGF1R inhibitor, suppresses growth and metastasis of breast cancer cells: SY-707 is an ALK/FAK/IGF1R inhibitor
Source: Acta Biochim Biophys Sin (Shanghai). 2022 Feb 10;54(2):252–60. doi: 10.3724/abbs.2022008 (PMC9909315; doi:10.3724/abbs.2022008)
Supplement: 446FigS1 [file 446FigS1.docx]

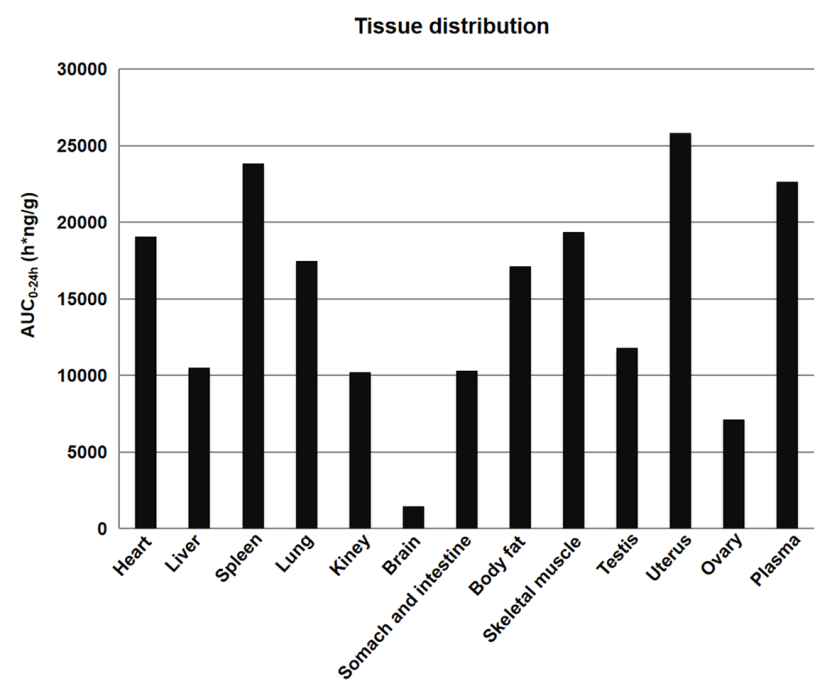


**Supplementary Figure S1. The tissue distribution profile of SY-707** Tissue distribution of SY-707 in SD rats was measured after oral administration of SY-707 (15 mg/kg).
